# The impact of species complexes on tree abundance patterns in Amazonia

**DOI:** 10.1002/ajb2.16069

**Published:** 2022-10-04

**Authors:** Christine D. Bacon, Adrian Hill, Hans ter Steege, Alexandre Antonelli, Gabriel Damasco

**Affiliations:** ^1^ Department of Biological and Environmental Sciences University of Gothenburg Carl Skottsbergs gata 22B SE‐413 19 Gothenburg Sweden; ^2^ Gothenburg Global Biodiversity Centre Box 461 SE‐405 30 Gothenburg Sweden; ^3^ Naturalis Biodiversity Center Leiden The Netherlands; ^4^ Royal Botanic Gardens, Kew TW9 3AE Richmond, Surrey UK; ^5^ Department of Plant Sciences University of Oxford South Parks Road Oxford OX1 3RB UK; ^6^ Departamento de Botânica e Zoologia Universidade Federal do Rio Grande do Norte Natal RN 59078‐970 Brazil

Amazonian forests are the largest and most diverse forests in the tropics. Interestingly, Amazonian tree species abundance is not evenly distributed, and most areas are inhabited by a few, highly abundant species, termed oligarchic (Pitman et al., 2001) or hyperdominant (ter Steege et al., 2013). In this essay, we suggest that for some species, hyperdominance can be a result of species complexes and poorly defined species. We examined genomic studies on different hyperdominant species, which together suggest that inconsistent taxonomy can bias our understanding of abundance patterns. If hyperdominant Amazonian trees actually consist of multiple biological species, estimates of their abundances would be affected, and Amazonian tree diversity may be even greater than previously proposed.

## THE PATTERN

Amazonia is a global center for plant diversity, with high species richness across spatial scales (Macía and Svenning, 2005). An early study on plant diversity in Amazonia revealed that relative abundances of species—a component of biodiversity that describes how common or rare a species is relative to other species—are highly variable among regions (Gentry, 1988). Much of our understanding of Amazonian tree diversity comes from standardized plot surveys of individuals over a certain diameter at breast height (e.g., ≥10 cm, Amazon Tree Diversity Network, Amazon Forest Inventory Network).

By fitting mathematical models to a relative abundance distribution, biological processes can be hypothesized from the model parameters, such as Fisher's log series. Fisher's log‐series distribution was fit to rank abundance data from across 1170 plots from across Amazonia to determine species abundance in the region (ter Steege et al., 2013; and across 1946 plots by ter Steege et al., 2020).

The current patterns of commonness, rarity, and richness in Amazonian tree diversity is based on 3.9 × 10^11^ individuals and an estimated ca. 16,000 species of trees. Further, ter Steege et al. (2013) found 227 species that they termed hyperdominant, which together account for half of all individual trees in Amazonia. Particular ecological factors and functional traits may be important, but the road to hyperdominance appears to be either being present everywhere or being locally dominant, such as in one or two regions of the major regions of Amazonia (e.g., northwestern and southwestern Amazonia; ter Steege et al., 2013).

However, another avenue to hyperdominance could be paved by artefacts, for example, due to under‐sampling. Here we provide our perspective on the consequences of species complexes on hyperdominance, which is increasingly being recognized as an important aspect of Amazonian biodiversity (e.g., Bacon et al., 2021).

## THE PROBLEM

The determination of hyperdominant species status relies on correct botanical identification of tree species in forest plots. While defining a species is potentially one of the most difficult tasks in biology and highly dependent on the species concept applied (de Queiroz, 2007), it is a fundamental unit of biological science. One major limitation to the correct identification of Amazonian trees is the scarcity of diagnostic features on herbarium vouchers, which often lack flowers and fruits, since many closely related tree species are difficult to distinguish based on vegetative traits alone; trees are tall and bear few or no flowers or fruits for most of the year (Goodwin et al., 2015). And although the use of DNA sequencing for species identification is now increasingly affordable and can even be done in near real time, most identifications are based on morphological characters. It is therefore reasonable to assume that botanical identification errors occur, for example, where rare taxa may be lumped with the local dominant taxon.

Taxonomy also changes through time and is a dynamic science. In Amazonia for example, in the most recent monograph of *Guatteria*, 62 species were put into synonymy with 15 other species (10 into *G. hirsuta* and 26 into *G. punctata*), and 10 new species were described, representing a net “loss” of 52 species (Mass et al., 2015). A contrasting example is *Astrocaryum* for which 18 species (Henderson et al., 1995) grew to over 40 in the most recent monograph (Kahn, 2008).

**Figure 1:**
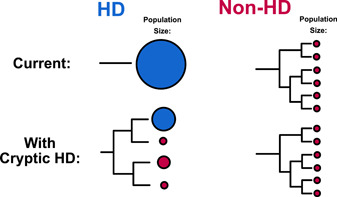
Hyperdominant (HD) versus non‐HD relative species diversity and population sizes currently and as proposed in this paper. Currently, HD species are few with massive population sizes (blue circles), while non‐HD species are many but with small population sizes (red circles). Here we propose that HD species may more frequently comprise species complexes containing multiple cryptic species. Some of these cryptic species may no longer be considered dominant based on the new population sizes compared to those currently considered nondominant.

Outside of these more technical issues in determining hyperdominant species, a suite of biological processes may function more often in hyperdominant than nonhyperdominant species, such as recent divergence and recurrent hybridization. Some hyperdominants may be complexes of insipient species, originating when there is diversification with little or no morphological change, morphological divergence coupled with little genetic divergence, or when only a subset of populations become morphologically and genetically distinct (Pinheiro et al., 2018). Population size plays an important role in these processes; as a fundamental unit of coalescence, effective population size determines the time it takes for lineages to coalesce into a most recent common ancestor. Large populations, like those of hyperdominants, may be easier to break up, but subsequent allopatric speciation takes longer (Figure 1).

In reviewing the literature for the 20 most abundant species in Amazonia (Table 1 in ter Steege et al., 2013), we found convincing evidence of the presence of species complexes within the most hyperdominant species. The strongest argument is found within the most abundant species in Amazonia, *Euterpe precatoria*. There are over 5.2 billion individual *E. precatoria* palm trees estimated across four different Amazonian regions (ter Steege et al., 2013), suggesting its hyperdominance is due to it being widely distributed. However, a recent genetic study of this species rejects this interpretation in favor of multiple genetically distinct entities encompassed by the species. Phylogenetic analysis of the genus identified a nonmonophyletic *E. precatoria*, with the two sampled individuals in the phylogeny separated by highly supported branches, where one is sister to *E. edulis* and the other to *E. broadwayi* (Pichardo‐Marcano et al., 2019). Dense population‐level sampling within *E. precatoria* strongly supports high population structure (Ferreyra Ramos et al., 2021) to the extent that most populations, even if adjacent, do not hybridize and could therefore be considered separate biological species (Barrerio Sánchez, 2013).

The 12th most abundant species in Amazonia, *Protium heptaphyllum*, provides further persuasive evidence that abundance patterns can be an artefact of constituent species complexes. Morphological, genomic, and functional data of *P. heptaphyllum* are consistent with eight separately evolving lineages, each warranting species status (Damasco et al., 2021), at least under a unified concept of species (De Queiroz, 2007). This strong evidence, however, contrasts with that shown for the third most abundant species, *Eschweilera coriacea*, where recent genetic analysis identified no conclusive evidence for cryptic species, at least within French Guiana (Heuertz et al., 2020).

## THE IMPLICATIONS

**Figure 2:**
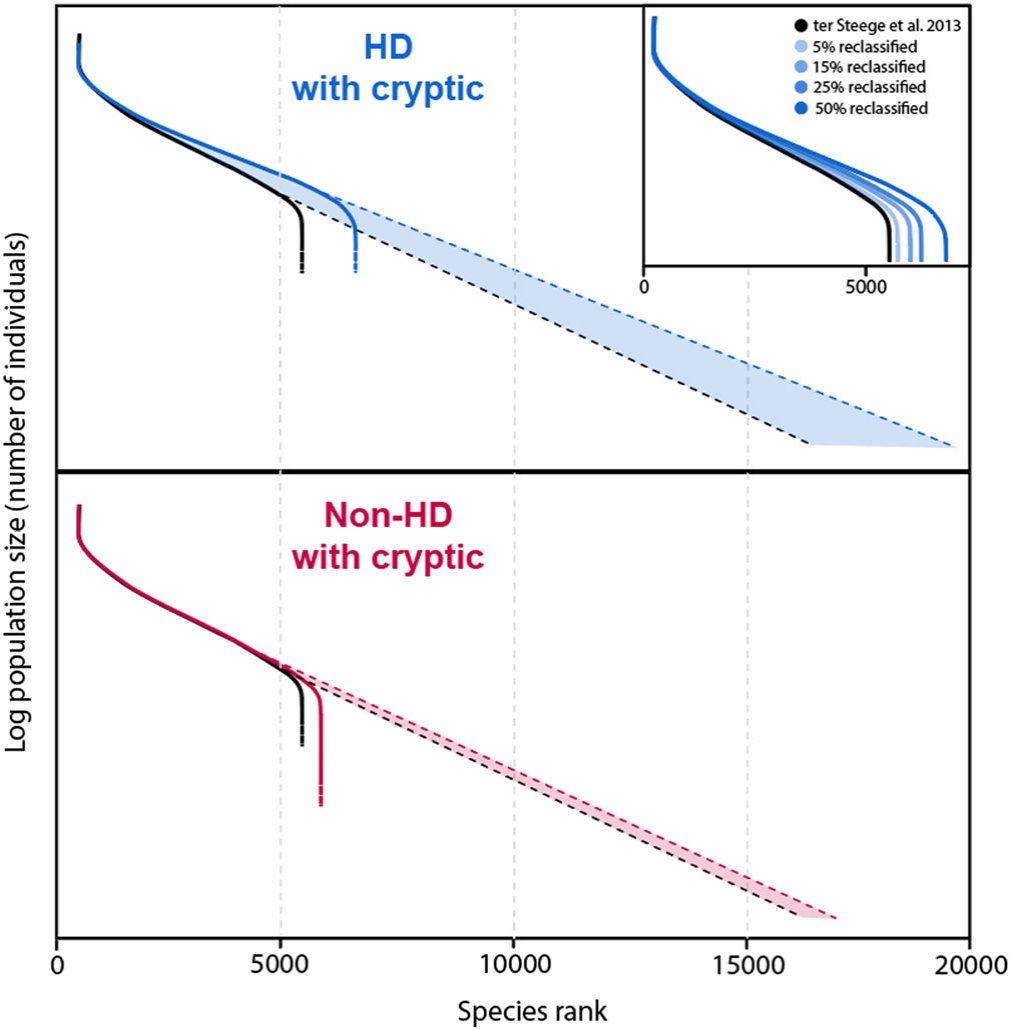
Rank abundance diagrams extrapolated to estimate the number of Amazonian tree species. Here both the number expected for hyperdominant (HD) and non‐HD species comprised of cryptic species complex(es) is shown with solid lines. Linear extrapolations (dashed lines) that yield the estimated number of species for Amazonia change dramatically when considering that half of the 227 hyperdominant species (ter Steege et al., 2013) are reclassified into multiple taxa (inset). The number of possible new species for each reclassified taxon and their relative abundances were randomized. The black curve is the rank abundance distribution of ter Steege et al. (2013).

If species are poorly defined and actually constitute cryptic species complexes, the relative abundance distribution of Amazonian plant diversity would present a flatter distribution curve than currently inferred (Figure 2). The ultimate implication is that there are many more species expected in Amazonia than the 10,071 species currently recognized (ter Steege et al., 2019).

In the future, we suggest using species delimitation tools that include genomic data such as those implementing coalescent approaches across the geographic distribution of hyperdominant species, not only to explicitly test species boundaries, but also to make more reliable botanical identifications, particularly in documented or suspected species complexes. We cannot protect what we do not know and having precise taxonomy together with conservation assessments would allow for the recognition of potentially unknown threatened taxa and geographic regions for potential conservation priority.

In addition, genomics can contribute to the understanding of ecosystem functioning, particularly if newly recognized taxa show differential responses to global change. Although potentially biased by unrecognized species complexes, evidence suggests that hyperdominant species have similar ecological functions as nondominant species but are more resilient to change, providing stability to ecosystems (Walker et al., 1999). Some hyperdominant species were found to have wider niche breadths on average than rare species in the same communities, which also suggests high adaptive potential to environmental variation (Heuertz et al., 2020). This potential could be fundamental to overcoming pressures related to climate change, especially since Amazonian hyperdominant species were shown to be more sensitive to climate than their Andean counterparts (Arrellano et al., 2014). Future directions for the understanding of hyperdominant species includes modeling genomic vulnerability to climate and land‐use change. Analyses of genome–environmental associations can also be coupled with deforestation projections for extinction risk modeling.

Understanding species diversity and abundance patterns accurately is essential for the conservation and management of Amazonian forests. As the largest source of lineages to other biomes on the continent, the forests of Amazonia have played a fundamental role in the origin and evolution of neotropical biodiversity (Antonelli et al., 2018). The benefits hyperdominant species provide to people are wide, and life in many tropical communities is based on these species for construction materials and food security. This legacy must continue if indeed most of the biogeochemical cycling in the world's largest tropical forest is performed by a small proportion of its diversity.

## AUTHOR CONTRIBUTIONS

C.D.B.: Conceptualization; Writing–Original Draft Preparation. A.H.: Investigation; Writing–Review & Editing. H.t.S.: Conceptualization; Writing–Review & Editing. A.A.: Conceptualization; Writing–Review & Editing. G.D.: Conceptualization; Investigation; Writing–Review & Editing.
